# Kidney tubular function and serum phosphate levels in HIV-1-infected patients treated with tenofovir: preliminary results

**DOI:** 10.1186/1758-2652-13-S4-P83

**Published:** 2010-11-08

**Authors:** P Grima, A Zizza, P Calabrese, R Chiavaroli, A Faraone, M Tana, MR Sodo, P Tundo, M Guido

**Affiliations:** 1 S. Caterina Novella Hospital, Division of Infectious Diseases, HIV Centre, Galatina, Italy; 2 National Research Council, Institute of Clinical Physiology, Lecce, Italy; 3 S. Caterina Novella, Via Roma, Galatina, Italy;; 4 University of Salento, Di.S.Te.B.A., Faculty of Sciences, Lecce, Italy

## Purpose of the study

There is concern that human immunodeficiency virus (HIV) infection and the use of highly active antiretroviral therapy lead to cumulative toxicity. Tenofovir (TDF) is the first choice for most subjects. Even if it has a safe metabolic profile, much attention has been fixed on kidney tubular function and regulation of phosphate metabolism. We performed this study to evaluate the role of a TDF based regimen has on renal tubular over time.

## Methods

Prospective, cross-sectional, single centre study was carried out. 121 HIV-1-infected patients were consecutively enrolled in six groups based on duration of TDF exposition: G0, from 6 to 12 months; G1 from 13 to 24 months; G2 from 25 to 36 months; G3 from 37 to 48 months; G4 more than 48 months and G5 under HAART but never exposed to TDF. Glomerular function was assessed using creatinine clearance (CrCL) calculated by MDRD. Tubular function was assessed using fractional excretion ratio of phosphate and normalized renal threshold phosphate concentration. Demographic, CD4, serum phosphate levels, viral load were collected.

## Summary of results

A total of 121 consecutive HIV-1-infected patients were analyzed: 15 in G0, 11 in G1, 14 in G2, 32 in G3, 35 in G4 and 14 in G5. Mean of TDF exposure was 10.26, 21.4, 36.2, 47.3 and 67.4 months in G0, G1, G2, G3 and G4 respectively. There was no statistically significant difference of mean values of FEP(11.2, 10.3, 8.4, 9.8, 11.1 and 10% in G0, G1, G2, G3, G4 and G5 respectively), TmPO4/GFR (3.5, 3.5, 3.6, 3.6, 3.4 and 3.4 mg/dl in G0, G1, G2, G3, G4 and G5 respectively ), CrCL (102.2, 94.3, 92.9, 106.5, 103.1 and 101.6 ml/min/1.73m2 in G0, G1, G2, G3, G4 and G5 respectively) and serum phosphate levels (3.4, 3.3, 3.1, 3.5, 3.3 and 3.4 in G0, G1, G2, G3, G4 and G5 respectively) between groups. Moreover, we did not find correlation of FEP (r:0.04, p:0.6) and TmPO4/GFR (r:0.05, p:0.5) with duration of TDF therapy.

## Conclusions

Treatment with TDF is not associated with altered kidney tubular function and serum phosphate levels over time.

